# Defective Autophagy in Atherosclerosis: To Die or to Senesce?

**DOI:** 10.1155/2018/7687083

**Published:** 2018-02-26

**Authors:** Mandy O. J. Grootaert, Lynn Roth, Dorien M. Schrijvers, Guido R. Y. De Meyer, Wim Martinet

**Affiliations:** ^1^Laboratory of Physiopharmacology, University of Antwerp, Antwerp, Belgium; ^2^Department of Immunohistochemistry, HistoGeneX, Antwerp, Belgium

## Abstract

Autophagy is a subcellular process that plays an important role in the degradation of proteins and damaged organelles such as mitochondria (a process termed “mitophagy”) via lysosomes. It is crucial for regulating protein and mitochondrial quality control and maintaining cellular homeostasis, whereas dysregulation of autophagy has been implicated in a wide range of diseases including atherosclerosis. Recent evidence has shown that the autophagic process becomes dysfunctional during the progression of atherosclerosis, regardless of whether there are many autophagy-stimulating factors (e.g., reactive oxygen species, oxidized lipids, and cytokines) present within the atherosclerotic plaque. This review highlights the recent insights into the causes and consequences of defective autophagy in atherosclerosis, with a special focus on the role of autophagy and mitophagy in plaque macrophages, vascular smooth muscle cells (VSMCs), and endothelial cells (ECs). It has been shown that defective autophagy can promote apoptosis in macrophages but that it accelerates premature senescence in VSMCs. In the ECs, defective autophagy promotes both apoptosis and senescence. We will discuss the discrepancy between these three cell types in their response to autophagy deficiency and underline the cell type-dependent role of autophagy, which may have important implications for the efficacy of autophagy-targeted treatments for atherosclerosis.

## 1. Introduction

Atherosclerosis is a chronic inflammatory disease which is characterized by the formation of lipid-containing plaques in the vessel wall of large- and medium-sized arteries [1, 2]. They preferentially develop at branching points where blood flow is low/disrupted and the barrier function of the endothelial cell (EC) layer is compromised. Besides their lipid-rich content, atherosclerotic plaques consist of vascular smooth muscle cells (VSMCs), inflammatory cells (e.g., macrophages, dendritic cells, T lymphocytes, and mast cells) and extracellular matrix (ECM) [1, 2]. Atherosclerotic plaque stability is maintained by the formation of a thick fibrous cap overlaying a large necrotic core of oxidized lipids and necrotic debris derived from VSMCs and macrophages. The thick fibrous cap is formed by numerous VSMCs producing large amounts of ECM, such as collagen, elastin, and proteoglycans. As the plaque progresses, the number of inflammatory macrophages increases and the necrotic core enlarges, while the fibrous cap becomes thinner due to increased VSMC death and ECM degradation [3]. Destabilization of atherosclerotic plaques increases the incidence of plaque rupture and the subsequent occlusion of arteries, leading to acute coronary syndrome, myocardial infarction, and stroke [2, 4]. Risk factors of atherosclerosis include hypertension, hypercholesterolemia, diabetes mellitus, obesity, and smoking [5], but also aging is considered to be an important contributing factor to this disease [6]. According to epidemiological studies, the incidence and prevalence of atherothrombotic diseases, including myocardial infarction and stroke, increase with advancing age [7]. Moreover, atherosclerosis itself is an important risk factor for the development of abdominal aortic aneurysm (AAA) that can lead to a high level of mortality upon AAA rupture and subsequent bleeding [8]. In fact, the vulnerable atherosclerotic plaque shows similar pathological features with the aortic aneurysm wall including VSMC apoptosis, ECM remodeling, and macrophage-mediated inflammation [9, 10], though whether the association between atherosclerosis and AAA is truly causal or rather a result of common risk factors is still unclear [8, 11]. Hence, despite the application of cholesterol-lowering therapies (statins), surgical interventions (balloon angioplasty and stenting), and lifestyle changes (diet and physical exercise), atherosclerosis remains the leading cause of death in the developed world [12]. In search of new therapies to prevent these life-threatening complications, further exploration of the different processes involved in plaque destabilization and rupture is necessary. Recent evidence indicates that (dysfunctional) autophagy plays an important role in atherosclerotic plaque destabilization and the overall development of the disease [13–16].

Autophagy is an evolutionarily conserved subcellular process which is involved in the degradation of proteins and damaged organelles in lysosomal compartments. Different types of autophagy (macroautophagy, microautophagy, and chaperone-mediated autophagy) have been identified, dependent on the delivery route of the cargo to the lysosomal lumen [17]. In this review, we will focus on macroautophagy (further referred to as autophagy), including mitophagy, a highly selective form of macroautophagy that specifically targets damaged mitochondria.

The autophagic process is characterized by the engulfment of cytoplasmic material in double-membraned vesicles, termed autophagosomes. Fusion of autophagosomes with lysosomes results in the formation of single-membraned autolysosomes. The incorporated material is then degraded into amino acids, carbohydrates, fatty acids, and nucleotides upon exposure to lysosomal hydrolases [18]. Through the recycling of biomolecules, basal autophagy provides new building blocks to support cellular function and homeostasis. However, when cells are exposed to intracellular (e.g., accumulation of damaged organelles) or extracellular (e.g., nutrient deprivation, hypoxia) stressful stimuli, autophagy is triggered and serves as a cell survival mechanism [19]. In case of damaged mitochondria, mitophagy is initiated through P-TEN-induced kinase (PINK1) and Parkin activation. Upon loss of mitochondrial membrane potential, PINK1 accumulates at the outer membrane where it recruits Parkin, facilitating the autophagic degradation of dysfunctional mitochondria [20]. However, unrestrained and excessive stimulation of autophagy can lead to autophagic cell death, as a result of the degradation of vital cellular components [21].

The nucleation and elongation of autophagosomes are directed by specific autophagy-related (ATG) proteins such as ATG5 and ATG7. These proteins are in turn regulated by multiple upstream signaling factors, such as the mammalian target of rapamycin (mTOR) and AMP-activated protein kinase (AMPK). Of all the pathways, the nutrient-sensing mTOR pathway has been investigated the most extensively. In conditions of nutrient deprivation, mTOR is inactivated to allow the autophagy-mediated generation of new building blocks and energy supplies. In case of nutrient excess, autophagy becomes inactivated upon mTOR activation [22]. AMPK is situated upstream of mTOR and its activation results in mTOR inhibition and induction of autophagy [23].

In this review, we will discuss the role of autophagy in atherosclerosis, with a special focus on the impact of defective autophagy on plaque macrophages, VSMCs, and ECs.

## 2. Autophagy in Atherosclerosis

In the past decade, multiple lines of research have provided evidence for the occurrence of autophagy in human and experimental atherosclerosis. Using transmission electron microscopy (TEM), it has been shown that autophagy can occur in all major cell types (i.e., macrophages, VSMCs, and ECs) of human atherosclerotic plaques [24, 25]. The autophagic cells are characterized by the incorporation of amorphous material in cytosolic vacuoles, which are distinct from lipid droplets and lysosomes. They are mainly localized in the fibrous cap and near the necrotic core but at relatively low frequencies (approximately 1.5%), which is similar to the degree of apoptosis. The morphological evidence for autophagy has been further supported by Western blot and immunohistochemical analyses of advanced human plaques showing elevated levels of the autophagosomal marker LC3-II [13, 25]. Moreover, analysis of human VSMCs isolated from carotid plaque specimens shows increased mitophagy, including elevated levels of PINK1 as compared to normal healthy VSMCs [26]. Nevertheless, comparison of carotid plaques from symptomatic versus asymptomatic patients revealed a 5-fold decrease in LC3-II expression [27], suggesting a decline in autophagy in relation to the clinical disease stage. Furthermore, analyses of atherosclerotic plaques from human and mouse samples have demonstrated the accumulation of p62 and polyubiquitinated proteins that colocalize with plaque macrophages [28]. The expression of p62 in murine atherosclerotic plaques has also been shown to be further elevated with age and plaque burden [29]. In human carotid artery plaques, p62 expression has been shown to be increased in maximally versus minimally diseased regions and shows strong colocalization with polyubiquitin but not with LC3 [30]. Given its role in targeting polyubiquitinated proteins to the autophagosome for degradation, increased levels of p62 in atherosclerotic plaques likely reflect dysfunctional autophagy. In fact, defects in the autophagic machinery can occur at two different stages: (1) defects during the initiation stage, that is, the formation/maturation of the autophagosomes or (2) defects during the fusion stage with the lysosomes and/or the lysosomal-mediated degradation. It is thought that defective autophagy in atherosclerosis is mainly due to defects in the lysosomal-dependent pathway. Indeed, autophagy in advanced plaques plays a role in the formation of ceroid, an insoluble complex of proteins and oxidized lipids that accumulates in lysosomes [31]. To degrade the engulfed ceroid, hydrolytic enzymes are distributed to the ceroid-containing lysosomes, and therefore, they cannot participate in active autolysosomes any longer [32]. Moreover, extensive or persistent oxidative stress can disrupt lysosomal membrane integrity, leading to the release of lysosomal hydrolases and the inadequate removal of damaged mitochondria by mitophagy. As a result, cytochrome c is released from the damaged mitochondria and may promote apoptosis [33, 34]. Finally, cholesterol crystals accumulating in advanced atherosclerotic plaques can damage the lysosomal membrane, undermining the autophagic process [35]. These findings suggest that the lysosomal-mediated degradation of engulfed cytoplasmic material can be disrupted in advanced atherosclerosis, rather than the initiation of autophagy itself. Thus, autophagy may still become activated in advanced lesions, in response to autophagy inducers (e.g., ROS, oxidized lipids, and ER stress) present in the plaque, but the process becomes dysfunctional during the second stage which depends on the lysosomal-dependent degradation of the cargo. Therefore, the function of the lysosomal system, and in particular the role of transcription factor EB (TFEB), which is a master regulator of lysosomal biogenesis and autophagy, has become an important topic of investigation in the field of atherosclerosis and other lipid metabolism-related disorders [30, 36–39]. Besides defects in the autophagic machinery, autophagy may become insufficient, especially in advanced lesions where high amounts of oxidative stress have accumulated. In this scenario, autophagy is functional but then fails to cope with the excessive amount of stress in the plaque, leading to cell apoptosis [40]. Hence, the cytoprotective autophagic pathway may turn into a maladaptive pathway, depending on the developmental stage of the plaque.

## 3. Role of Autophagy in Macrophages, Vascular Smooth Muscle Cells, and Endothelial Cells

In vitro experiments have shown that autophagy can be activated in macrophages, VSMCs, and ECs by different atherosclerosis-related stimuli.

**Figure 1:**
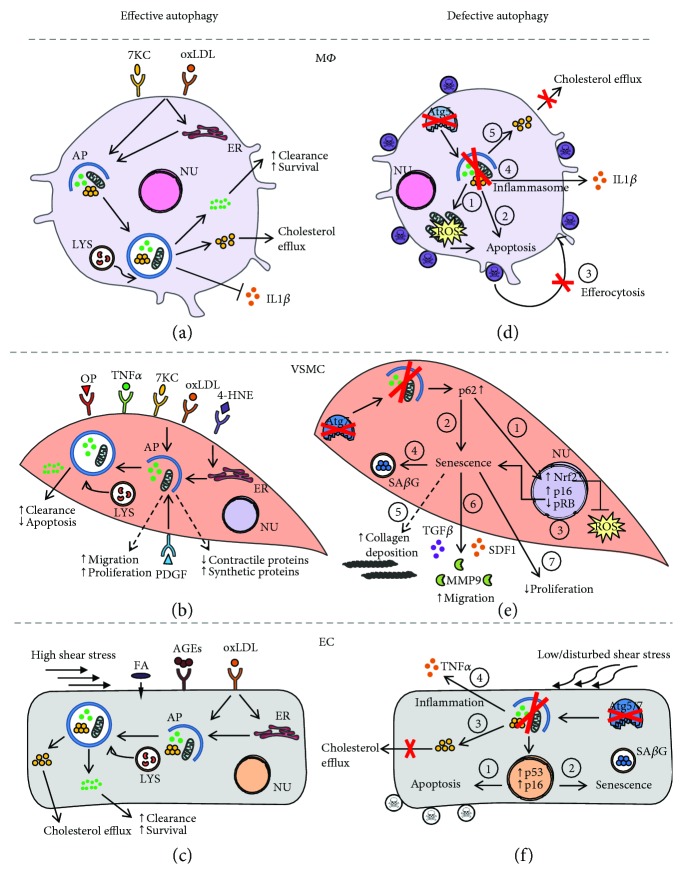
Role of autophagy in macrophages, vascular smooth muscle cells, and endothelial cells in atherosclerosis. (a) Oxidized lipids (e.g., oxLDL and 7-ketocholesterol) present in atherosclerotic plaques can stimulate autophagy in macrophages (M*Φ*) in either a direct manner or indirectly through induction of ER stress. Degradation of damaged proteins and organelles in the autophagosome favors cell survival. Specific removal of dysfunctional mitochondria by mitophagy also limits inflammation. Macrophage autophagy also promotes cholesterol efflux by regulating the delivery of lipid droplets to lysosomes. (b) Autophagy in vascular smooth muscle cells (VSMCs) can be triggered by various atherosclerosis-related stimuli such as oxLDL, 7-ketocholesterol, 4-hydroxynonenal, osteopontin, and TNF*α*. oxLDL activates mitophagy in VSMCs as a safeguarding mechanism against apoptosis. VSMC autophagy may also be stimulated by the growth factor PDGF that promotes the development of a synthetic, hyperproliferative VSMC phenotype. (c) Autophagy/mitophagy in ECs can be stimulated by different atherogenic stimuli such as oxLDL, AGEs, and saturated fatty acids to promote EC survival. Upon oxLDL exposure, autophagy is activated either directly or indirectly through induction of ER stress and facilitates oxLDL degradation. Also exposure to high shear stress stimulates protective autophagy in ECs. (d) Defective autophagy in macrophages (e.g., by *Atg5* deficiency) results in accumulation of damaged proteins and organelles, such as mitochondria, which leads to increased oxidative stress (1) and apoptosis (2). Apoptotic autophagy-deficient macrophages are not properly phagocytized (3). Autophagy-defective macrophages are further characterized by hyperactivation of the inflammasome (4) and impaired cholesterol efflux (5). (e) Defective autophagy in VSMCs (e.g., by *Atg7* deficiency) leads to accumulation of p62, resulting in activation of the Nrf2 antioxidative pathway (1) and p16/pRB-mediated cellular senescence (2). Autophagy-deficient VSMCs are characterized by cellular and nuclear hypertrophy (3), increased SA*β*G activity (4), collagen deposition (5), increased secretion of promigratory factors (TGF*β*, MMP9, and SDF1) (6), and decreased proliferation (7). (f) Exposure of ECs to low/disturbed shear stress impairs autophagy. Autophagy deficiency in ECs (e.g., by *Atg5* or *Atg7* deficiency) promotes apoptosis (1) and senescence (2). Autophagy-deficient ECs are also characterized by defective oxLDL degradation (3) and increased inflammation (4). 4-HNE: 4-hydroxynonenal; 7-KC: 7-ketocholesterol; AP: autophagosome; ER: endoplasmic reticulum; LYS: lysosomes; NU: nucleus; OP: osteopontin; ROS: reactive oxygen species; AGEs: advanced glycation end products.

In macrophages, autophagy can be stimulated by oxidized LDL (oxLDL) and 7-ketocholesterol, one of the primary oxysterols in oxLDL, either directly or indirectly via induction of ER stress (Figure 1(a)). Activation of autophagy promotes macrophage survival by facilitating the clearance of damaged proteins and organelles. Moreover, autophagy promotes the efflux of free cholesterol from macrophage foam cells by regulating the delivery of lipid droplets to lysosomes for degradation [41]. Mitophagy in macrophages can be stimulated by different inflammasome activators (e.g., lipopolysaccharide (LPS)) and requires p62 relocalisation to the mitochondria. Loss-of-function experiments have shown that p62-dependent clearance of damaged mitochondria can limit NF-*κ*B-mediated inflammation [42].

Similarly to macrophages, VSMC autophagy can be stimulated by oxidized lipids (Figure 1(b)) and promote cell survival [43, 44]. The lipid peroxidation product 4-hydroxynonenal (4-HNE) can activate autophagy in VSMCs [45] via the induction of ER stress to stimulate the removal of 4-HNE-modified proteins [46]. oxLDL has been shown to stimulate mitophagy as a safeguarding mechanism against VSMC apoptosis [34]. Exposure of human VSMCs to oxLDL triggers mitophagy, which is associated with accumulation of PINK1 and Parkin at the outer membrane of the damaged mitochondria [34]. In addition, VSMC autophagy may also be regulated by different cytokines (e.g., osteopontin and TNF*α*) [47] and growth factors such as the platelet-derived growth factor (PDGF). The latter induces VSMC autophagy via an AMPK- and mTOR-independent mechanism and protects VSMCs against 4-HNE-induced cell death [48]. Moreover, PDGF promotes the development of a synthetic VSMC phenotype, which is characterized by an enhanced migration and proliferation potential, decreased expression of contractile proteins, and upregulation of synthetic VSMC markers, indicating that autophagy may also regulate VSMC phenotype and proliferation [49].

Also in ECs, oxLDL has been shown to activate autophagy to convey protection against endothelial damage (Figure 1(c)) [50–52]. Upon oxLDL uptake, lipids are trafficked into autophagosomes for lysosomal-mediated degradation. However, oxLDL may also trigger autophagy through induction of ER stress. Splicing of the mRNA of X-box binding protein 1 (XBP1) triggers an autophagic response in ECs through transcriptional activation of Beclin 1, a key component in the autophagic machinery [53]. Also, proatherogenic factors such as advanced glycation end products (AGEs) trigger autophagy in ECs to protect against endothelial injury [54]. Furthermore, exposure of ECs to shear stress induced by laminar blood flow on the vessel wall has been shown to stimulate autophagy [55–57]. Of note, only high shear stress stimulates protective autophagy in ECs, while low shear stress causes a defect in endothelial autophagy as a result of mTOR activation and AMPK pathway inhibition [57]. Moreover, treatment of ECs with palmitic acid triggers PINK1-Parkin-mediated mitophagy to maintain mitochondrial quality control and prevent EC injury [58]. Besides its role in the regulation of EC survival, autophagy is also involved in many other EC functions such as NO production [59], angiogenesis [60], and thrombosis [61, 62].

To further define the role of autophagy in macrophages, VSMCs, and ECs, cell-type specific autophagy-deficient knockout mice have been generated. Deletion of the essential autophagy gene *Atg5* in macrophages results in enhanced ROS generation, increased apoptosis, and reduced efferocytosis [14] (Figure 1(d)). The latter suggests that apoptotic autophagy-deficient macrophages are poorly recognized and cleared by phagocytes [14]. Furthermore, autophagy deficiency in macrophages has also been shown to impair cholesterol efflux [41] and can lead to increased IL1*β* secretion upon treatment with LPS and cholesterol crystals [29], a well-known characteristic of NLR family pyrin domain containing 3 (NLRP3) inflammasome activation. Importantly, deletion of *Atg7* in macrophages causes similar effects [29], indicating that the observed changes in macrophage behavior are due to a general defect in autophagy, rather than defects in specific autophagy-related genes.

In contrast to macrophages, genetic deletion of *Atg7* in murine VSMCs does not induce apoptosis but triggers stress-induced premature senescence [16], a state of irreversible growth arrest whereby cells remain metabolically active and undergo multiple morphological and functional changes. For example, *Atg7*-deficient VSMCs develop cellular and nuclear hypertrophy, a p16/RB-mediated cell cycle arrest and senescence-associated *β*-galactosidase (SA*β*G) activity (Figure 1(e)) [16]. *Atg7*-deficient VSMCs also show increased collagen content, augmented migration potential, and elevated protein expression of the cytokine transforming growth factor beta (TGF*β*) and stromal cell-derived factor 1 (SDF1). According to RT-qPCR analysis, *Atg7*-deficient VSMCs show increased mRNA levels of interleukin 1 beta (IL1*β*) and NLRP3, which is indicative of inflammasome hyperactivation. Interestingly, the hyperbiosynthetic state of *Atg7*-deficient VSMCs does not trigger activation of the unfolded protein response (UPR), as evidenced by the lack of XBP1 splicing and unaltered expression of C/EBP homologous protein (CHOP) and phosphorylated eukaryotic initiation factor 2 alpha (P-eIF2*α*), indicating that autophagy deficiency in VSMCs does not evoke ER stress. All these findings are in line with the typical characteristics of human senescent VSMCs, as recently described by Gardner et al. [63], except for the observed increase in collagen content. Although senescence in mice can be regulated differently to humans [64, 65], we cannot exclude the possibility that the increased collagen content could be related to the activation of other cellular pathways, rather than being part of the senescent phenotype itself.

Impairment of autophagy in ECs promotes both apoptosis and senescence (Figure 1(f)) [57]. The aortas of mice deficient in endothelial *Atg5* or *Atg7* show increased EC senescence as evidenced by SA*β*G activity and p16-positive nuclei as compared to control mice. Similarly, inhibition of autophagy by a pharmacological inhibitor or genetic knockdown of *Atg5* induces senescence in HUVECs [57]. Also noteworthy is that the endothelial cell layer of the aortas of EC-specific *Atg5* knockout mice is characterized by the accumulation of TUNEL- and p53-positive cells, indicative of increased apoptosis. Autophagy deficiency in HUVECs also promotes inflammation and hampers the ability of the ECs to align with the direction of flow [57]. Furthermore, HUVECs lacking *Atg7* accumulate oxLDL at higher levels than autophagy-competent cells, underlining the critical role of EC autophagy in oxLDL degradation [52].

## 4. Consequences of Defective Autophagy in Atherosclerosis

As autophagy becomes disrupted during atherosclerosis progression, the understanding of its effects on plaque stability is imperative for the exploration of potential autophagy-targeting treatments for atherosclerosis.

It has recently been shown that macrophage-specific deletion of *Atg5* can accelerate atherosclerotic plaque development in mice lacking the LDL receptor (*LDLR^−/−^* mice) fed a Western-type diet (WTD) for 16 weeks [14]. Plaques from these mice develop an unstable phenotype as illustrated by an increase in necrotic core area, apoptosis, and defective efferocytosis [14]. Moreover, plaques from *Atg5*-deficient *LDLR^−/−^* mice show an increased accumulation of cholesterol crystals, which could promote hyperactivation of the NLRP3 inflammasome [29]. Impaired macrophage autophagy also stimulates macrophage polarization into a proinflammatory M1 phenotype [66]. Furthermore, defective macrophage autophagy exerts proatherogenic effects by impeding reverse cholesterol transport [67]. Thus, these findings indicate that defective macrophage autophagy can promote atherosclerotic plaque destabilization by interfering with multiple key processes including cholesterol transport, inflammation, ROS generation, and apoptosis.

VSMC-specific deletion of *Atg7* has been shown to accelerate atherosclerotic plaque development in WTD-fed ApoE lipoprotein-deficient (*ApoE^−/−^*) mice and to promote postinjury neointima formation [16]. VSMCs within the lesions are characterized by several senescence markers, including SA*β*G activity, RB hypophosphorylation, and nuclear hypertrophy. Injury-induced carotid neointimal lesions of *Atg7*-deficient mice show increased collagen deposition and upregulation of matrix metallopeptidase 9 (MMP9), TGF*β*, and SDF1 [16]. Interestingly, Gardner et al. showed that senescent human VSMCs also release active MMP9 and support the current view that VSMC senescence contributes to atherogenesis, at least partially via the establishment of a senescence-associated secretory phenotype [63]. All together, these findings indicate that autophagy-defective VSMCs can develop a senescence-associated secretory phenotype that conveys proatherogenic effects.

The loss of functional autophagy in ECs has been shown to accelerate atherogenesis in two independent studies. In the first study, *ApoE^−/−^* mice with an endothelial-specific *Atg7* deletion show an increase in plaque burden after 16 weeks of WTD [52]. Importantly, these mice show a marked increase in lipid deposition throughout the whole aorta, indicating that functional autophagy in ECs is critical for controlling lipid accumulation in the vessel wall [52]. In the second study, the impact of defective endothelial autophagy on atherosclerosis under physiological (high shear stress) and pathological (low shear stress) blood flow was examined. After 10 weeks of WTD, endothelial-specific deletion of *Atg5* in *ApoE^−/−^* mice leads to massive plaque development in the descending aorta, an area known to be atheroresistant, when compared to controls. However, plaque burden is not different between both groups in the atheroprone regions of the aortic arch, in which EC autophagy is likely already impaired upon exposure to low shear stress [57]. These findings indicate that functional endothelial autophagy limits plaque formation under high shear stress conditions, whereas deficiency in EC autophagy promotes atherosclerosis in these atheroresistant regions. Moreover, the high shear stress regions in *ApoE^−/−^* mice with an EC-specific *Atg5* gene deletion are characterized by increased EC apoptosis and senescence, two pathways known to favor atherogenesis.

## 5. Cell Type-Dependent Role of Autophagy

Although the consequences of defective autophagy in VSMCs, macrophages, and ECs are similar in terms of plaque progression, the mechanisms by which defective autophagy aggravates atherosclerosis in these cell types are different. Defective autophagy in macrophages promotes plaque instability through increased macrophage apoptosis and necrosis, while in autophagy-defective VSMCs, senescence contributes to accelerated plaque formation. The consequences of defective autophagy in ECs are even more complex, as deletion of essential autophagy genes leads to both apoptosis and senescence. The opposing responses of VSMCs, macrophages, and ECs to impaired autophagy raise several important questions:

### 5.1. Why Does Defective Autophagy Lead to Senescence in VSMCs?

To unravel the molecular link between defective autophagy and VSMC senescence, p62 was overexpressed in autophagy-competent VSMCs to mimic the accumulation of p62 observed in autophagy-defective VSMCs. Overexpression of p62 induces p16/RB-mediated senescence in autophagy-competent VSMCs [16], indicating that severe accumulation of p62 mediates the induction of senescence in VSMCs. Also in other pathologies (such as primary biliary cirrhosis and chronic obstructive pulmonary disease), it has been suggested that p62 can play a role in the induction of senescence [68, 69]. It is important to note that accumulation of p62 also leads to activation of the nuclear factor-erythroid 2-related factor 2 (Nrf2) and antioxidant responsive element (ARE) pathway in *Atg7*-deficient VSMCs, rendering them more resistant to oxidative stress-induced cell death as compared to autophagy-competent VSMCs [16]. Moreover, p62 accumulation in *Atg7*-deficient VSMCs is not only a result of defective p62 degradation but is also due to an increase in p62 mRNA expression. Indeed, a previous report by Jain et al. demonstrated that p62 is a target gene for Nrf2, creating a positive feedback loop between Nrf2 activation and p62 accumulation [70].

### 5.2. Is It Conceivable That Defective Autophagy Can Lead to Senescence?

According to the current literature, the relationship between autophagy and senescence is rather controversial. Several reports support a direct link [71–74] between these two pathways, while others favor an inverse relationship [75–81]. The latter is related to their opposing roles in aging; autophagy has been shown to promote cellular and organismal longevity [82], whereas senescence has been described as a central hallmark of the aging phenotype [83], notwithstanding both pathways share common characteristics such as vacuolization, responsiveness to stress, and cell preservation. When cells are exposed to internal (accumulation of damaged organelles) or external (nutrient deprivation, hypoxia) stressors, autophagy is activated to replenish nutrient supply, generate energy, and/or dismantle dysfunctional mitochondria. In case of senescence, cells escape from toxic or stressful insults by entering a prolonged growth arrest, but they remain metabolically active. Both autophagy and senescence pathways can thus be engaged as an attempt to evade cell death.

So far, the direct positive relationship between autophagy and senescence has only been reported for oncogene and DNA damage-induced senescence but not yet for stress-induced premature senescence and thus argues for a distinct role of autophagy in the different types of senescence. Nevertheless, it is still difficult to comprehend why autophagy, a process known for its lifespan-extending and antiaging properties, would take place in senescent cells.

The inverse relationship between autophagy and senescence seems conceivable when they are both considered as cytoprotective pathways. Autophagy has been postulated as an adaptive response mechanism to countermeasure cellular senescence [84–87]. By facilitating the removal of damaged mitochondria and protein aggregates, autophagy limits the accumulation of oxidative and proteotoxic stress, respectively, and could therefore negatively regulate cellular senescence. In this regard, defects in the autophagic process could accelerate the development of senescence. Multiple reports have shown that the expression of autophagic markers are reduced in aged tissues, whereas tissue-specific knockout of autophagy-related genes leads to the development of premature aging phenotypes in different mouse models (reviewed in [82]). Although the evidence is mostly circumstantial, these studies suggest that dysfunctional autophagy is not only associated with aging but could also actively contribute to the aging phenotype. Conversely, stimulation of autophagy by the mTOR inhibitor rapamycin has been shown to suppress senescence [79–81].

### 5.3. Why Does Defective Autophagy Not Lead to Senescence in Macrophages?

Research in our laboratory has demonstrated that macrophages with impaired autophagy do not develop cellular senescence [16]. The reason for the absence of senescence in autophagy-defective macrophages is still unclear, although some hypotheses have been suggested. One possible hypothesis is related to the intracellular levels of p62. In contrast to VSMCs, *Atg7*-deficient macrophages do not show increased expression of p62 at the mRNA level. Accordingly, relatively low levels of p62 in autophagy-defective macrophages may not be able to either induce senescence or activate the Nrf2-ARE pathway. Nevertheless, the loss of p62 in macrophage-specific *Atg5*-deficient *LDLR^−/−^* mice does not improve but actually exacerbates the proatherogenic phenotype of these mice [28]. Plaques of these double-knockout mice are characterized by increased apoptosis, necrosis, inflammation, and accumulation of insoluble polyubiquitinated proteins, when compared to p62-competent mice [28]. The authors suggest that the increased atherogenesis is linked with increased inflammation, due to the defective p62-dependent clearance of inflammasomes, recently described as “inflammasomophagy” [88].

A second hypothesis is that the response of cells to stress seems to be predetermined by their origin. In general, hematopoietic and germ cells tend to respond to stress by undergoing apoptosis, whereas mesenchymal cells (e.g., fibroblast-like cells) tend to respond to stress by engaging the senescence program [89]. Hence, the dissimilarity between VSMCs and macrophages in their responses to defective autophagy could simply lie in their origin.

A third hypothesis includes the cell's capacity to proliferate and to renew. One would expect that highly proliferative cells would be more prone to senesce in response to stress, though the opposite seems to be true. Rapidly dividing cells, such as hematopoietic and germ cells, undergo apoptosis rather than senescence upon telomere shortening [90]. It is clear that this is still an area of investigation that requires further experimentation, and we hope that our review article will stimulate further work in this area.

### 5.4. Why Does Defective Autophagy Lead to Apoptosis in Macrophages?

The increased susceptibility for apoptosis in autophagy-deficient macrophages is likely linked to the observed increase in cellular ROS production [14]. According to previous studies, the loss of essential autophagy proteins is frequently associated with an increased accumulation of dysfunctional mitochondria and ROS [91–93] and often leads to (apoptotic) cell death [94–97]. These findings are in line with the role of autophagy as a housekeeping and cytoprotective mechanism. In response to ROS, autophagy is activated to restore homeostasis by limiting oxidative damage and removing dysfunctional mitochondria. In this way, defects in the autophagic pathway can easily lead to ROS accumulation which, in turn, can trigger apoptotic cell death. Furthermore, the autophagic removal of dysfunctional mitochondria by mitophagy prevents apoptosis by reducing the release of cytochrome c and other proapoptotic factors [34, 98]. In cases of nutrient depletion, mitochondria undergo mitochondrial membrane permeabilization (MMP), but the autophagy-dependent supply of endogenous nutrients avoids cell death [97]. When autophagy is suppressed, however, the bioenergetic state of the cell reaches an absolute minimum, while mitochondria undergoing MMP are no longer removed, forcing the cell to undergo apoptosis [97]. Overall, autophagy is considered to be a major countermeasure to oxidative stress and apoptosis, and thus any form of dysregulation of this pathway can facilitate these processes, which are known contributors to atherosclerotic plaque destabilization.

### 5.5. Is It Conceivable That Defective Autophagy Leads to both Apoptosis and Senescence in Endothelial Cells?

In contrast to macrophages and VSMCs, ECs undergo both apoptosis and senescence in response to impaired autophagy. Considering the cytoprotective role of autophagy in ECs, defects in the endothelial autophagic machinery are likely to cause apoptosis. For example, inhibition of autophagy abolishes the protection against H_2_O_2_-induced apoptosis [55]. Also, exposure to an excess of oxLDL impedes the protective autophagic response via upregulation of the LOX-1 scavenger receptor and triggers apoptotic EC death [99]. In vivo evidence confirmed that vascular ECs in EC-specific *Atg5* knockout mice show an increased incidence of apoptosis. However, the observed increase in p53-positive nuclei may not be exclusively linked to apoptosis, as p53 is a core component of the acute DNA damage response pathway that initiates senescence. Interestingly, ECs have been shown to acquire a proapoptotic phenotype when reaching senescence [100–102]. Indeed, senescent ECs are more sensitive to apoptotic stimuli such as TNF*α*, oxLDL, and ceramide than their younger counterparts, which likely result from reduced levels of NO and increased ROS-induced damage [100–102]. As such, senescence and apoptosis are complementary, rather than opposing, cell fates in autophagy-impaired ECs. Given that adequate autophagy is essential for preserving proper EC function and that endothelial dysfunction contributes to arterial aging [103], we hypothesize that defective endothelial autophagy plays a direct role in aging-related arterial dysfunction. Nevertheless, additional mechanistic studies are necessary to determine how defective autophagy promotes EC senescence.

## 6. Potential Pitfalls for the Detection of Autophagy and Senescence

Autophagy can be monitored via different methods such as transmission electron microscopy, Western blotting, and immunohistochemistry. Each of these techniques has its advantages and limitations. However, without knowledge of the difficulties and drawbacks of these assays, the results could easily be misinterpreted (reviewed in [104, 105]). Additionally, some factors need to be taken into consideration when investigating the autophagic process during senescence. Especially the interpretation of senescence-associated *β*-galactosidase (Sa*β*G) staining as a marker of senescence can be quite challenging. SA*β*G is a lysosomal enzyme, which is typically expressed by senescent cells and that has an optimal activity at pH 6, likely reflecting the increase in lysosomal mass during senescence [106]. Experiments using lysosomal inhibitors need to be interpreted carefully as they can block autophagic flux by increasing lysosomal pH and can therefore falsely increase the level of SA*β*G. Moreover, autophagy induction is generally characterized by an increase in lysosomal size, as a result of membrane fusion with the autophagosome [107, 108] and can thus influence SA*β*G staining. The use of the autophagy inhibitor 3-methyladenine in chemotherapy-induced senescence is precarious as it can potentiate chemotherapy-induced apoptosis [109] and thereby interfere with the incidence of senescence. Additionally, upregulation of the p53 pathway may not be an exclusive marker of apoptosis but could indicate senescence.

## 7. Pharmacological Stimulation of Autophagy in Atherosclerosis

In this review, we have described how autophagy becomes dysfunctional during atherosclerotic plaque progression and how this affects plaque stability. With this in mind, we recommend stimulation of autophagy as a plausible treatment strategy for atherosclerosis. The statin family of cholesterol-lowering drugs, which are notorious for their “pleiotropic effects” (such as anti-inflammatory effects, improvement of EC function, and plaque stabilization) [110], have been shown to stimulate autophagy in VSMCs, at least partially through inhibition of the mTOR pathway [111, 112]. More recently, antisenescence effects have been added to their list of actions. Statins prevent senescence in ECs and VSMCs, most likely due to their ability to limit telomere shortening and oxidative telomere DNA damage [113].

Besides statins, different autophagy-stimulating compounds are currently being tested as potential plaque-stabilizing therapies [15, 114]. One of the most promising compounds to date is rapamycin (or rapamycin derivatives (e.g., everolimus), termed rapalogs) that stimulates autophagy via mTOR inhibition. Apart from their known inhibitory effect on VSMC proliferation in drug-eluting stents, these mTOR inhibitors have been shown to elicit a cluster of antiatherosclerotic effects, including depletion of plaque macrophages, improvement of cholesterol efflux, lowering systemic and local inflammation, and inhibiting intraplaque neovascularization [115]. Moreover, mTOR inhibitors have been demonstrated to suppress cellular senescence and to prolong lifespan in a variety of animal species [116]. These promising data are slightly overshadowed by their adverse effects on blood glucose and lipid levels, so that combined therapy with metformin or statins is advisable. Moreover, it should be noted that continuous administration of everolimus in mice results in mTOR overactivation and tolerance and leads to a decrease in autophagy, at least in the liver tissue [117]. Therefore, an intermittent dosing regimen is recommended to exploit its full stimulatory effect on autophagy. It is important to note that some, but not all, beneficial effects of mTOR inhibition are related to autophagy induction. Some favorable effects of mTOR inhibitors are solely based on inhibition of cell proliferation.

The antidiabetic drug metformin stimulates autophagy and inhibits mTOR indirectly via activation of AMPK. Metformin has been proven to reduce micro- and macrovascular complications in diabetic patients [118], which likely goes beyond its hypoglycemic effect. Metformin suppresses vascular senescence [119] and inflammation [120] and has been shown to attenuate atherosclerosis in diabetic [121] and nondiabetic *ApoE^−/−^* mice [119, 122]. Metformin also improves mitochondrial quality control by reducing mitochondrial fragmentation in an AMPK-dependent manner [121]. Although recent work in our laboratory demonstrated that the anti-inflammatory effect of metformin on ECs is autophagy dependent [122], more studies are needed to determine whether the beneficial cardiovascular effects of metformin are linked to autophagy induction.

Because mitophagy is essential in atherosclerosis, new pharmacological compounds are needed that specifically target this process. Activation of autophagy by mTOR inhibition could be sufficient to remove damaged mitochondria, though this remains a nonselective way of promoting mitophagy [123]. Modulation of mitochondrial fission (i.e., fragmentation) or fusion may be a more valuable strategy for targeting mitophagy [123]. For example, treatment with the dynamin-related protein 1 inhibitor mdivi-1 reduces mitochondrial fragmentation and attenuates atherosclerosis in diabetic *ApoE^−/−^* mice [121]. However, the particular impact on mitophagy was not investigated in this study. Treatment with specific mitochondria-targeted antioxidants might be an alternative approach to maintain mitochondrial quality control [123]. Although these agents may not improve the elimination of damaged mitochondria, they prevent accumulation of mtROS and could thus limit mitochondrial damage and improve mitophagy [124].

## 8. Conclusion

Over the past few years, autophagy has emerged as a novel therapeutic target for the treatment of atherosclerosis. Multiple lines of evidence have suggested that autophagy becomes dysfunctional during the progression of atherosclerosis; thus, the investigation of its consequences on plaque stability has been of great importance. Interestingly, genetic disruption of the autophagic process can evoke opposing effects in plaque macrophages, VSMCs, and ECs. Macrophages respond to autophagy impairment by undergoing apoptosis, while VSMCs engage the senescence program, though both phenomena have been shown to promote plaque progression. In this regard, autophagy can be defined as a sensitive control switch between senescence and apoptosis. When autophagy is impaired, the cell has two main choices: either die (via apoptosis) or adapt (via senescence). However, autophagy-defective ECs do not favor one particular cell fate but can undergo both apoptosis and senescence. The reason for these cell type-specific differences remains poorly understood but may involve the accumulation of the linker molecule p62 or may simply depend on the cell's origin and/or proliferative capacity. Nevertheless, our findings suggest a significant role for p62 in VSMC aging and hopefully encourage others to focus on the involvement of p62 in EC senescence and other age-related processes. Hence, for the treatment of atherosclerosis, we favor an autophagy-stimulating approach, although one should be aware that unexpected cell type-dependent effects may occur that could challenge the application of autophagy-stimulating therapies. Moreover, excessive induction of autophagy could trigger autophagy-associated cell death; therefore, appropriate dosing would be advisable. The development of pharmacological approaches to specifically investigate mitophagy has only just begun; but given its significance in mitochondrial quality control and aging, we reason that they will be very valuable in the future treatment of cardiovascular disease. Based on the newly attained insights, we would favor to focus future research onto the development and testing of autophagy-/mitophagy-stimulating drugs, preferentially those with antisenescence potential. We consider drugs that have such a dual action highly promising not only for the treatment of atherosclerosis but also for age-related cardiovascular diseases in general.
